# From adhesion to biofilm: strain-specific and environmental factors shaping *Streptococcus mutans* biofilms

**DOI:** 10.1016/j.bioflm.2026.100391

**Published:** 2026-08-17

**Authors:** Chen Sun, Danuta Mazurel, Jingmei Yang, Antonius J.M. Ligtenberg, Dongmei Deng

**Affiliations:** a Department of Preventive Dentistry, Academic Centre for Dentistry Amsterdam, University of Amsterdam and Vrije Universiteit Amsterdam, Amsterdam, the Netherlands; b State Key Laboratory of Oral Disease and National Clinical Research Center for Oral Diseases, Department of Periodontics, West China Hospital of Stomatology, Sichuan University, Chengdu, China; c Department of Oral Biochemistry, Academic Centre for Dentistry Amsterdam, University of Amsterdam and Vrije Universiteit Amsterdam, Amsterdam, the Netherlands

**Keywords:** Salivary agglutinin, Laminar flow, Multispecies community, BioFlux™, Calgary biofilm device, Strain-level diversity

## Abstract

*Streptococcus mutans* is a key pathogen in dental caries and adheres to tooth surfaces via the surface protein SpaP, which binds salivary agglutinin (SAG). However, the extent to which adherence properties influence subsequent biofilm formation remains unclear. This study investigated biofilm formation of *S. mutans* strains with varying adherence properties on glass surfaces with or without a crude SAG (cSAG) coating prepared from human parotid saliva, under static or laminar flow conditions, and in mono- or multispecies conditions. Green-fluorescent protein-labeled *S. mutans* strains (V403, NG8, C67-1, and UA159) were tested using the Calgary Biofilm device (CBD) and the BioFlux™ microfluidic system. Following a 2 h adhesion phase and 10 h of biofilm development, adherence and biofilm formation were quantified using resazurin assay or image analysis. cSAG coating enhanced adherence and biofilm formation under flow in strains V403, NG8, and C67-1, whereas UA159 showed low adherence and almost no biofilm formation under flow. All strains formed substantially more biofilm under flow than under static conditions, with increases exceeding 200-fold for V403 and NG8. Under laminar flow, the presence of a multispecies community generally reduced the amount of *S. mutans* in biofilms. In conclusion, cSAG coating promoted both adherence and biofilm formation, particularly under flow conditions. However, variation in initial cSAG-mediated adherence among *S. mutans* strains did not predict subsequent biofilm formation. Instead, biofilm formation was influenced by multiple factors, including strain-specific characteristics, flow conditions, and microbial community composition.

## Highlights

• SAG coating enhances *S. mutans* biofilm formation under laminar flow in all strains.
• Multispecies community inhibits *S. mutans* biofilm formation in most strains.
• *S. mutans* biofilm formation is shaped by strain traits and environment.
• For a specific strain, adherence does not correlate with biofilm formation capacity.

## 1 Introduction

Bacterial biofilms are structured microbial communities in which cells adhere to surfaces and to one another within a self-produced extracellular matrix [1]. This mode of growth enhances bacterial survival under environmental stresses and is associated with a wide range of infections [2]. Biofilm formation is commonly described as a multistep process consisting of five stages: (1) initial bacterial adherence to a surface; (2) irreversible attachment, accompanied by the production of extracellular polymeric substances (EPS); (3) bacterial growth and early biofilm architecture development leading to microcolony formation; (4) maturation of the biofilm structure; and (5) dissemination of individual cells from the mature biofilm [3]. Among these stages, initial adherence is considered a pivotal step, and strategies that inhibit bacterial attachment have been proposed as effective means to limit biofilm development [4].

However, it is important to note that several studies have shown that early attachment does not consistently correlate with final biofilm formation [5,6]. For instance, variations in surface properties and growth conditions can significantly influence biofilm architecture in *Escherichia coli*, despite similar levels of initial adhesion [7]. Similarly, no direct association has been observed between adhesion-related genes and biofilm-forming ability in *Staphylococcus aureus* strains [8]. These findings suggest that biofilm development could be influenced by additional environmental and biological factors beyond initial adhesion, particularly in complex host-associated ecosystems such as the oral cavity.

Dental caries is a widespread disease of the tooth hard tissues caused by sugar-driven acid production within dental biofilms, leading to demineralization and tooth decay. It remains a major global health burden affecting populations worldwide. *Streptococcus mutans,* a key contributor to dental caries, is well-known for its ability to adhere to surfaces and form biofilms [9]. The adherence of *S. mutans* is mediated by both sucrose-dependent and sucrose-independent mechanisms [9]. Sucrose-dependent adhesion involves glucosyltransferases that convert sucrose into extracellular glucans, forming a matrix that promotes aggregation and stable biofilm formation [8]. In contrast, sucrose-independent adhesion is mediated by salivary agglutinin (SAG), a high-molecular-weight mucin-like glycoprotein containing multiple scavenger receptor cysteine-rich (SRCR) domains that serve as binding targets for the *S. mutans* surface adhesin SpaP [7,10], enabling *S. mutans* attachment and aggregation in the absence of sucrose [11].

Notably, SAG-mediated adhesion exhibits substantial strain-dependent variation in *S. mutans*, which is associated with structural differences in SpaP [12]. In addition, oral environmental factors, including salivary flow and interactions with other microbial species, may further influence both adhesion efficiency and subsequent biofilm formation [13]. Despite these insights, it remains unclear how these strain-specific properties and ecological factors collectively modulate sucrose-independent adhesion and its contribution to biofilm development under physiologically relevant, multispecies conditions.

This study aims to investigate biofilm formation of *S. mutans* strains with distinct adherence properties to SAG coated surfaces under varied environmental conditions, including static and flow settings and in the presence of saliva-derived microbiota. Based on our previous publication [12], four *S. mutans* strains representing different adherence levels were selected for this study: high-adherence strains (V403 and NG8), a medium-adherence strain (UA159) and a low-adherence strain (C67-1). Biofilms were cultured using two biofilm model systems: the Calgary Biofilm Device (CBD), which contains 96 polystyrene pegs, and the microfluidic BioFlux™ system, which enables biofilm growth under defined laminar flow conditions.

## 2 Material and methods

### 2.1 Bacterial strains and culture condition

Four *S. mutans* strains UA159 (laboratory strain), V403 (clinical isolate), NG8 (laboratory strain) and C67-1 (clinical isolate) [14] were routinely cultured on Brain Heart Infusion (BHI) agar or in BHI broth at 37 °C under anaerobic condition (80% N_2_, 10% CO_2_, 10% H_2_). For experiments using the BioFlux™ system, strain carrying the *E. coli*-*S. mutans* shuttle vector pDM35 was used. pDM35 is a derivative of pVA838 [15] and contains a constitutive P32 promoter fused to a *gfpmut2* gene, resulting in constitutive green fluorescent protein (GFP) production. Erythromycin at 10 μg/ml was added to BHI broth or biofilm growth medium (BM) to maintain plasmid selection. The BM was prepared as described by Exterkate et al. [16] and contained 2.5 g/l mucin (M2378 Sigma), 2.0 g/l Bacto peptone (Difco 0118-01-8), 2.0 g/l Trypticase peptone (BBL 211921), 1.0 g/l yeast extract (Bacto 212750), 0.35 g/l NaCl, 0.2 g/l KCl, 0.2 g/l CaCl 2, 0.001 g/l hemin (Sigma H1652), and 0.0002 g/l vitamin K1, and 15.1 g/l PIPES at pH 7.0.

### 2.2 Saliva collection and processing

Two types of saliva were collected for the experiments. Parotid saliva from a single healthy volunteer was used to extract crude SAG (cSAG), whereas pooled unstimulated saliva from 6 healthy volunteers served as inoculum for bacterial adhesion and biofilm formation. Parotid saliva collection and cSAG extraction were performed following the procedures described by Yang et al. [12]. Briefly, saliva was collected using a Lashley cup, kept on ice immediately after collection for 30 min, and centrifuged at 4,000xg for 10 min at 4 °C. The resulting pellet was resuspended in sterile Milli-Q water to obtain cSAG for subsequent experiments. Unstimulated saliva was collected after volunteers abstained from oral hygiene for at least 24 h and from food and drink for at least 2 h, then pooled, diluted in 60% glycerol, and stored at −80 °C until use.

All volunteers were systemically and orally healthy, with no active caries or periodontal disease. Written informed consent was obtained, and the study was approved by the Ethical Committee of the faculty (document numbers 201962 and 2021-40362).

### 2.3 Adherence and biofilm formation of *S. mutans* in CBD

**Fig. 1:**
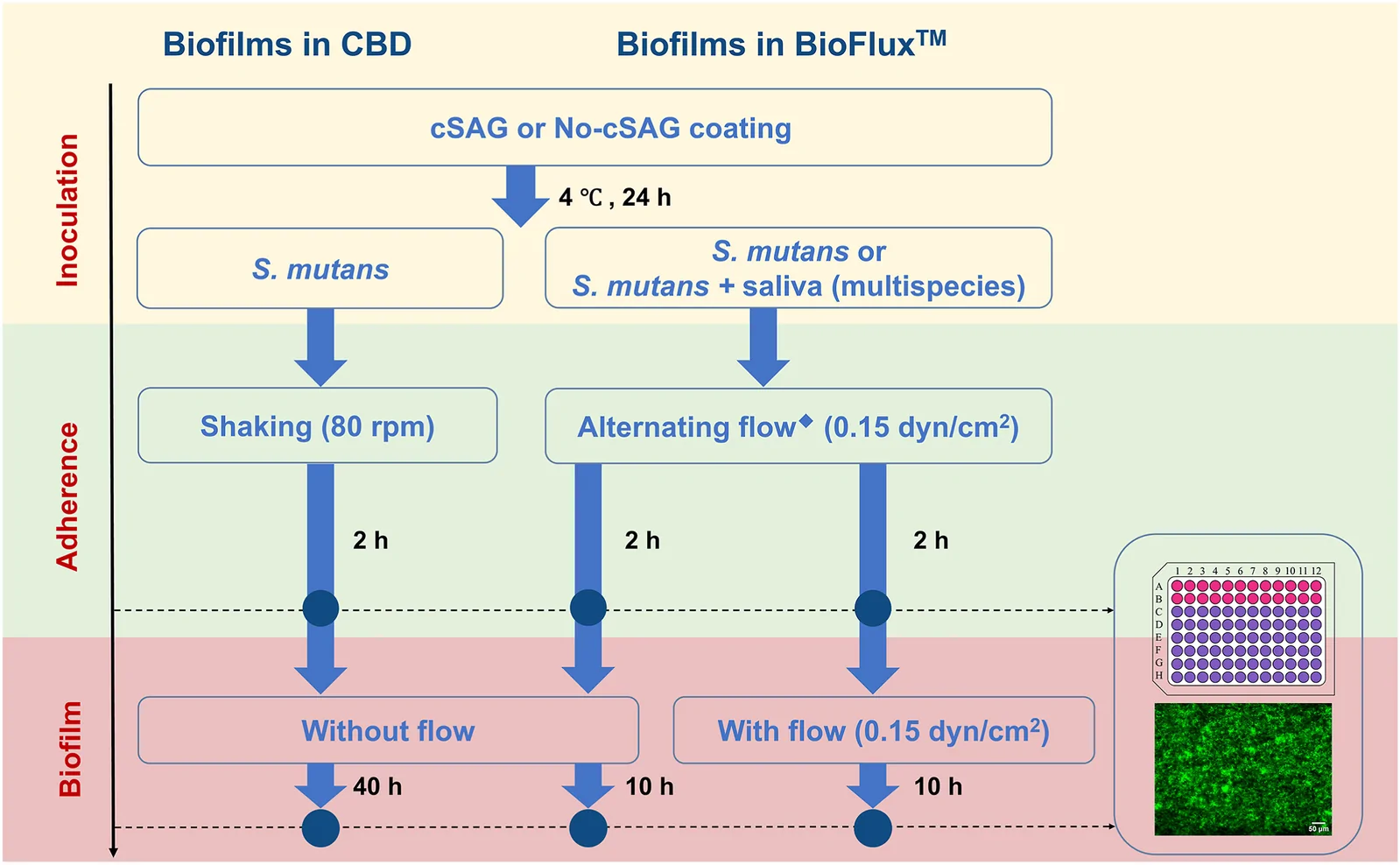
**Flowchart illustrating experimental procedures for adherence and biofilms using Calgary Biofilm Device (CBD) and BioFlux^TM^.** Adherence and biofilm were conducted in adherence buffer and biofilm growth medium, respectively. ● refers to measurement or imaging time points. Adherence and biofilm in CBD and BioFlux™ were quantified using resazurin assays and imaging acquisition, respectively. **^◆^** refers to alternating forward and reverse flow for 10 s every 20 min cSAG, crude salivary agglutinin. No-cSAG, coating buffer only.

The adherence and biofilm formation of four *S. mutans* strains on pegs with or without cSAG coating were first assessed using the CBD and subsequently evaluated in the BioFlux™ microfluidic system. Fig. 1 illustrates the overall experimental workflow.

#### 2.3.1 CBD model

cSAG was diluted 1:4 in coating buffer (0.1 M sodium carbonate, pH 9.2). Either the diluted cSAG or coating buffer alone was added to a 96-well plate at 200 μL/well. The plate was then covered with a lid containing polystyrene pegs (Nunc™, Roskilde, Denmark) and incubated overnight at 4 °C. Prior to the adherence step, the pegs were washed with phosphate-buffered saline containing 0.1% Tween 20 (PBST) to remove non-specifically bound proteins.

Adherence of *S. mutans* to coated pegs was performed as described previously [12]. Briefly, mid-log phase *S. mutans* cells were washed and resuspended in adherence buffer (3.73 g/l KCl, 0.11 g/l CaCl_2_, 0.01 g/l MgCl_2_, 0.14 g/l KH_2_PO_4_, pH 6.0) to a density of 3x10^9^ CFU/ml. Cell resuspensions (200 μL/well) were added to a new 96-well plate at and covered with the lid containing coated pegs. The plate was incubated for 2 h under anaerobic condition at 37 °C shaking at 80 rpm. The shaking condition was introduced to minimize the retention of weakly attached bacterial cells, allowing evaluation of more stable bacterial adhesion [17,18]. The amount of *S. mutans* adhered to the pegs was assessed using a resazurin assay.

After the adherence assay was completed, the pegs were washed with PBS and transferred to a new 96-well containing 200 μL/well of BM, with or without 0.2% sucrose, to allow subsequent biofilm development under anaerobic condition at 37 °C without shaking. BM was refreshed twice daily, at 8 and 16 h. After 40 h, biofilm formation on the pegs was quantified by the resazurin assay.

The experiment with CBD was independently performed 4 times for each strain, with 3 biological replicates per condition.

#### 2.3.2 Resazurin assay

Pegs were washed once with PBS and transferred to 0.0016% resazurin solution for 2h at 37 °C [19]. Fluorescence intensity (FI) of the resazurin solutions was measured using a spectrofluorometer (Spectramax M2, Molecular Device) with excitation at 485 nm and emission at 580 nm. A resazurin solution without bacteria served as the negative control for all measurements. The FI of each sample was corrected by subtracting the FI of the negative control before further calculations.

### 2.4 Adherence and biofilm formation of *S. mutans* in BioFlux™

A BioFlux 1000Z microfluidic system (Fluxion Biosciences, San Francisco, CA, USA) integrated with an Axio Observer Z1 inverted microscope (Zeiss, Jena, Germany) and a monochrome CCD-camera (Exi Aqua Bio, QImaging, Surrey, Canada) was used for automated shear flow experiments. This setup was used to assess the adherence of *S. mutans* strains, alone or combined with a pooled saliva inoculum, to surfaces with or without cSAG coating, as well as subsequent biofilm formation under laminar flow or static conditions.

#### 2.4.1 cSAG coating in BioFlux™ system

Microchannels within a BioFlux 48-well plate (Fluxion Biosciences) were first primed by adding 200 μL coating buffer to the inlet well and applying a flow of 0.5 dyn/cm^2^ for 5 min to fill the channels and eliminate air bubbles. Subsequently, channels were filled with either 300 μL cSAG diluted in the same buffer or coating buffer alone (No-cSAG control), introduced through the inlet at 0.5 dyn/cm^2^ for 50 s. The plate was then incubated overnight at 4 °C to allow surface coating with cSAG.

#### 2.4.2 Adherence and biofilm formation

Coated channels were washed by introducing 500 μL of PBST into the inlet and applying a flow of 0.5 dyn/cm^2^ for 5 min to remove unbound cSAG, after which the channels were filled with adherence buffer using the same conditions. Mid-log phase (OD_600_ = 0.5) *S. mutans* cells were resuspended in either adherence buffer or diluted unstimulated saliva inoculum.

Serial dilutions of *S. mutans* suspensions were plated on Brain Heart Infusion agar plates, whereas those of the saliva inoculum were plated on Tryptic Soy agar containing 5% sheep blood, 5 μg/ml hemin, and 1 μg/ml menadione. The final cell density of *S. mutans* was approximately 3 x10^8^ CFU/ml, and the total bacterial count in the diluted saliva inoculum was approximately 8x10^6^ CFU/ml. Multiple colony morphologies could be observed in the saliva inoculum.

For each *S. mutans* strain, 500 μL of the bacterial suspension was added to the outlet well of an individual channel and backflowed at 0.5 dyn/cm^2^ until the channel was filled. The plate was then incubated for 2 h at 37 °C under anaerobic conditions to allow initial bacterial adherence. To promote active attachment during this period, laminar shear stress was applied intermittently by alternating forward and reverse flow at 0.15 dyn/cm^2^ for 10 s every 20 min. This alternating flow was used to provide a mechanical challenge comparable to the shaking condition applied in the CBD assay.

After the 2-h adherence period, adherence buffer was flowed through the channels at 0.5 dyn/cm^2^ for 2 min to remove non-adherent cells, after which BM (500 μL in the inlet well) was introduced into the channels by applying a flow of 0.15 dyn/cm^2^. Bacterial adherence was recorded by imaging. Biofilm formation was then monitored by real-time fluorescence and brightfield imaging every 30 min for 10 h. Two biofilm growth conditions were established: (1) flow condition: BM was continuously supplied at 0.15 dyn/cm^2^; (2) statistic condition: no flow was applied. The continuous laminar flow was selected to provide a controlled flow environment supporting biofilm development through continuous nutrient supply and waste removal.

In addition, for *S. mutans*-only groups without cSAG coating, BM was supplemented with 0.2% sucrose to assess sucrose-dependent biofilm development. Biofilm formation under this condition was also monitored by real-time imaging every 30 min for 10 h.

The experiment with BioFlux™ was independently performed 3 times for each strain, with 2 biological replicates per condition.

#### 2.4.3 Imaging acquisition

To acquire images, 6 regions of interest (ROIs) were selected along each channel, evenly distributed across its 6 mm length. The channel width (350 μm) corresponded to the image height. Fluorescence and brightfield images (10× objective) of each ROI were captured once for bacterial adherence or in real time for biofilm formation using the BioFlux Meta Imaging Series Software Version 7.8.1 (Molecular Devices, Downingtown, PA, USA). Fluorescence images were obtained using a FITC filter (Filter set 44, Zeiss) with excitation at 488 nm (bandwidth 30 nm) and emission at 520 nm (bandwidth 40 nm). Exposure times were 600 ms for adherence samples and 150 ms for biofilm samples. Brightfield images were acquired without excitation or emission filters, using an exposure time of 50 ms.

### 2.5 Image processing and quantification

All fluorescence images were analyzed using BiofilmQ Version 0.2.2 [20], a software platform for quantitative analysis of spatially structured biomass. Image analysis followed a three-step workflow: image preparation, segmentation and quantification. First, raw images were converted into BiofilmQ-compatible TIFF format. Next, bacterial biomass was segmented from the background using BiofilmQ. Finally, quantitative parameters were extracted using a 20 × 20 pixel analysis window, corresponding to an area of approximately 6.72 × 6.72 μm^2^, to assess *S. mutans* adherence and biofilm formation.

Biofilm_SubstratumCoverage (Coverage) was used to quantify the proportion of the surface covered by bacterial biomass. For adherence, this parameter was calculated from fluorescence images acquired after 2 h, and for biofilm formation from images acquired after 10 h.

Biofilm cluster count was determined based on the Cube_Volume_Fraction (Fraction) parameter obtained from BiofilmQ segmentation. This parameter represents the fraction of each analysis window occupied by bacterial biomass, with values approaching 1 indicate high biomass occupancy and dense cell regions, whereas lower values indicate more sparsely distributed cells. Analysis windows with a Fraction value of 1 were defined as biofilm clusters, and the number of these clusters within the analyzed image area was counted as the biofilm cluster count.

GridID and TrackID were used for time-series analysis. In BiofilmQ, each voxel in the segmented images, containing bacterial biomass, is assigned a unique GridID based on its x and y coordinates, which identifies its fixed position within the image. To trace the growth and detachment of bacterial clusters over time, voxels that are part of the same cluster are linked across frames using TrackID, while their GridIDs provide positional information. Together, these parameters allow each cluster to be followed as a coherent object over time, enabling quantification of the dynamics of individual bacterial clusters, including their growth, detachment, and spatial expansion.

### 2.6 Statistical analysis

The data were analyzed using GraphPad Prism 10.1.1. For the parameters Coverage and Fraction, measurements from six ROIs within a channel were summed to generate a single sample value representing that channel.

Two-way ANOVA was performed separately for each dependent variable: FI (from the resazurin assay), Coverage and Fraction (both from BiofilmQ image analysis), with cSAG coating, co-culture condition, or strain type as independent factors. Post-hoc (Tukey) tests were conducted to compare groups, and statistical significance was defined as p < 0.05.

## 3 Results

### 3.1 Adherence and biofilm formation of *S. mutans* strains in CBD

**Fig. 2:**
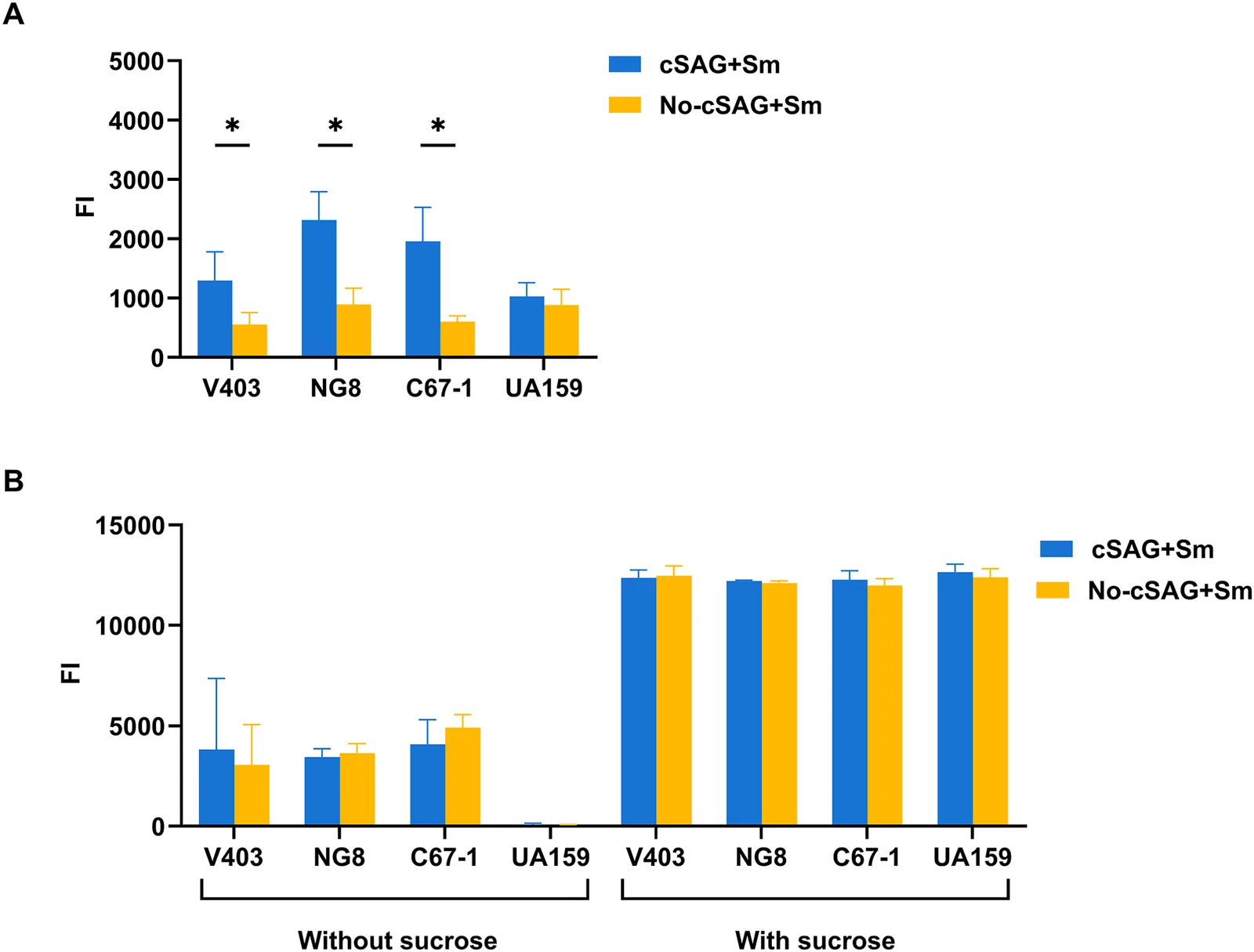
**Adherence and biofilm formation of *S. mutans* (Sm) strains in the Calgary Biofilm Device (CBD).** Adherence of *S. mutans* strains V403, NG8, C67-1, and UA159 to pegs coated with or without cSAG was measured after 2 h, and subsequent biofilm formation over 40 h was assessed in the presence or absence of 0.2% sucrose. Both adherence (**A**) and biofilm formation (**B**) were quantified using the resazurin assay. cSAG, crude salivary agglutinin. No-cSAG, coating buffer only. FI, fluorescence intensity. * refers to *P* < 0.05.

Strain-dependent adhesion and biofilm formation of *S. mutans* were first examined using CBD (Fig. 2). cSAG coating significantly increased 2-h adhesion of strains V403, NG8, and C67-1 compared with uncoated surfaces. In contrast, UA159 exhibited very low adhesion on cSAG-coated surfaces, comparable to those observed on uncoated surfaces (Fig. 2A).

cSAG coating did not significantly affect biofilm formation in any of the tested *S. mutans* strains (Fig. 2B). In the absence of sucrose, V403, NG8, and C67-1 produced comparable biofilm biomass regardless of cSAG coating, whereas UA159 did not form detectable biofilms. Supplementation with 0.2% sucrose markedly increased biofilm formation in all strains, including UA159, but this increase remained independent of cSAG coating.

### 3.2 Adherence and biofilm formation of *S. mutans* strains in BioFlux™

**Fig. 3:**
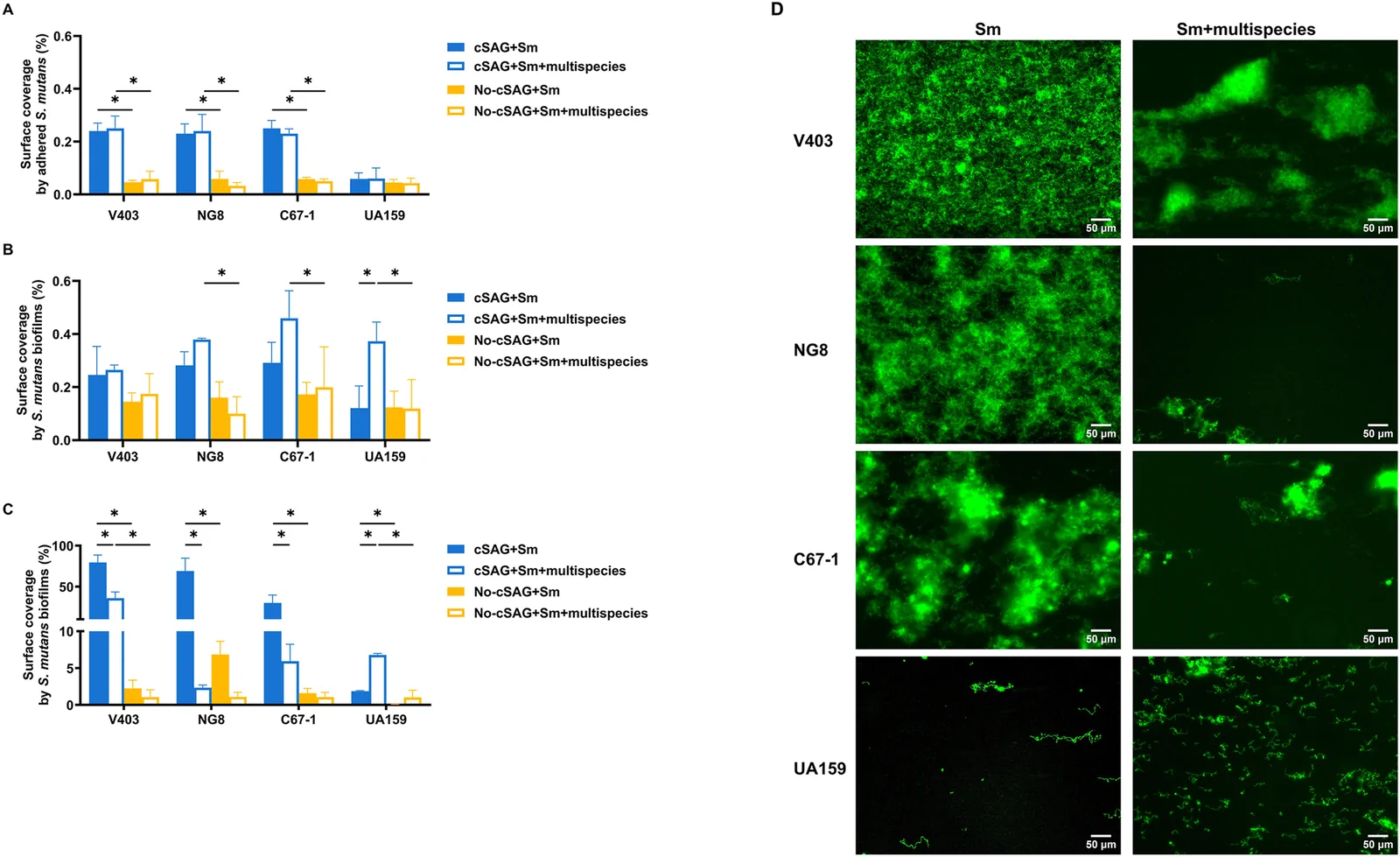
**Adherence and biofilm among *S. mutans* (Sm) strains in BioFlux^TM^ model.** (**A**) Adherence (2h) in alternating forward and reverse flow condition. It was measured from images by calculating the percentage of the area covered per channel. (**B**) Biofilm formation (10h) in static condition. It was measured from images by calculating the percentage of the area covered per channel. (**C**) Biofilm formation (10h) with laminar flow. It was measured from images by calculating the percentage of the area covered per channel. (**D**) Representative images of biofilm formation on cSAG coated surfaces under laminar flow at 10 h. Images were acquired using a 10× objective and a FITC filter (Filter set 44, Zeiss). Excitation was at 488 nm (bandwidth 30 nm) and emission at 520 nm (bandwidth 40 nm), with a 150 ms exposure time for all groups. Scale bar: 50 μm. cSAG, crude salivary agglutinin. No-cSAG, coating buffer only. * refers to P < 0.05.

Because CBD does not allow examination of biofilm formation dynamics, BioFlux™ was used to test the influence of cSAG coating, flow, and the presence of a multispecies community on *S. mutans* adherence and biofilm formation (Fig. 3).

Fig. 3A summarizes image analysis results after the initial 2-h adhesion of GFP-labeled *S. mutans* strains to surface with or without cSAG coating, in the presence and absence of multispecies culture. Consistent with the CBD results, cSAG coating significantly enhanced adhesion of strain V403, NG8, and C67-1, whereas UA159 showed low adhesion on both coated and uncoated surfaces. The presence of multispecies culture derived from a saliva inoculum did not alter the overall adhesion pattern.

Because the 40-h CBD biofilm assay was performed under static conditions, we first tested static conditions in BioFlux, monitoring biofilm formation for 10 h. Fig. 3B shows the percentage surface coverage at 10 h. In contrast to the adhesion phase, cSAG coating generally had little effect on biofilm formation: surface coverages of all strains were similar on coated and uncoated surfaces. In addition, the multispecies culture significantly increased biofilm formation by UA159 on cSAG-coated surfaces. C67-1, NG8 and UA159 showed significantly higher biofilm formation on cSAG-coated compared with uncoated surfaces (p < 0.05).

Application of laminar flow during biofilm development substantially altered biofilm formation among strains (Fig. 3C and D). Under flow, biofilm formation by V403, NG8, C67-1, and UA159 increased approximately 10-300-fold compared with static conditions, reaching 80%, 69%, 30% and 1.8% surface coverage within 10 h, respectively, but only on cSAG-coated surfaces. Without cSAG coating biofilm development was significantly less with 2%, 7%, 2%, and 0.1% surface coverage. Unlike static conditions, the presence of a multispecies culture generally inhibited biofilm formation on cSAG-coated surfaces, except for UA159, where biomass was higher in the presence of multispecies.

**Fig. 4:**
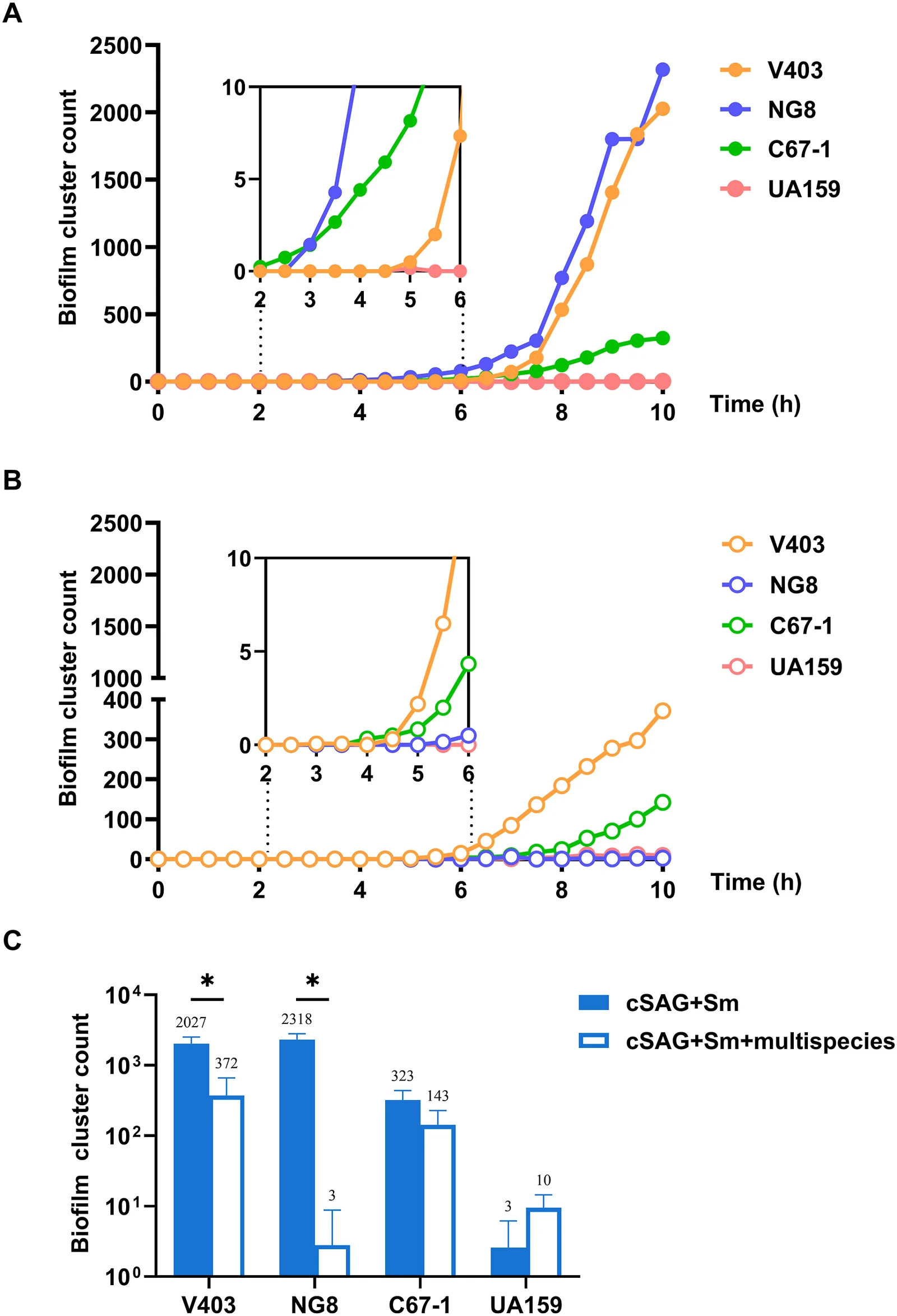
**Biofilm cluster formation count among *S. mutans* (Sm) strains in BioFlux model with crude salivary agglutinin (cSAG) with flow.** (**A**) Time-lapsed biofilm cluster counts within 10 h without multispecies. The inset shows zoom of the plot from 2 h to 6 h initial formation. (**B**) Time-lapsed biofilm cluster counts within 10h with multispecies. The inset shows zoom of the plot from 2 h to 6 h initial formation. (**C**) Biofilm cluster counts at 10h time-point. * refers to *P* < 0.05.

In addition, the application of 0.2% sucrose on biofilm development for each strain over a 10-h period was evaluated. For this examination, biofilm formation was initiated after cell adherence on uncoated surfaces under laminar flow. Unlike CBD biofilms formed under the same sucrose concentration, UA159 formed significantly less biofilm compared with the other 3 strains (Fig. 4 and Fig. S1 in Supplement).

### 3.3 Strain-dependent cluster formation in time under laminar flow

Given the strong effect of laminar flow on biofilm formation on cSAG-coated surfaces, biofilm development over time in 4 strains was further examined under this condition.

Fig. S2 (Supplement) shows representative images captured at 1, 3, 7, and 10 h, illustrating strain-specific biofilm architecture under flow. Under single-species conditions, V403 formed many small clusters, NG8 formed fewer but relatively larger clusters, and C67-1 formed the largest yet scattered clusters. In contrast, UA159 did not form discrete clusters and instead primarily formed long chains. In the presence of a multispecies culture, effects were evident within the first hour after flow initiation: V403 formed fewer but larger clusters and NG8 was largely washed out by 10 h. In contrast, UA159 surface coverage increased, although it remained low at 10 h, it was clearly higher than that observed for UA159 alone.

Fig. 4 further characterizes the dynamic biofilm development of different strains by quantifying biofilm cluster formation over time. Cluster counts were determined based on analysis regions of approximately 6.72 × 6.72 μm^2^ (45.2 μm^2^) that were fully occupied by biomass, as identified by BiofilmQ.

At 10 h, cluster counts generally followed the same strain-dependent trends observed for surface coverage (Fig. 3C). Under single-species conditions, V403 and NG8 showed substantially higher cluster counts than C67-1, while multi-species culture reduced cluster formation in V403 and NG8 but increased it in UA159 (Fig. 4C).

In addition to endpoint cluster counts, cluster formation over time showed some strain-specific variation in the timing of cluster appearance. NG8 and C67-1 initiated cluster formation earlier, whereas V403 showed a delayed onset (Fig. 4A inset). Multi-species culture further altered the timing of cluster formation, with delayed cluster initiation in NG8 and C67-1, whereas this was unaffected in V403 (Fig. 4B inset).

### 3.4 Strain-specific biofilm displacement over time under laminar flow

TrackID time-series analysis was used to assess displacement of individual *S. mutans* cells or clusters over time in the direction of flow.

**Fig. 5:**
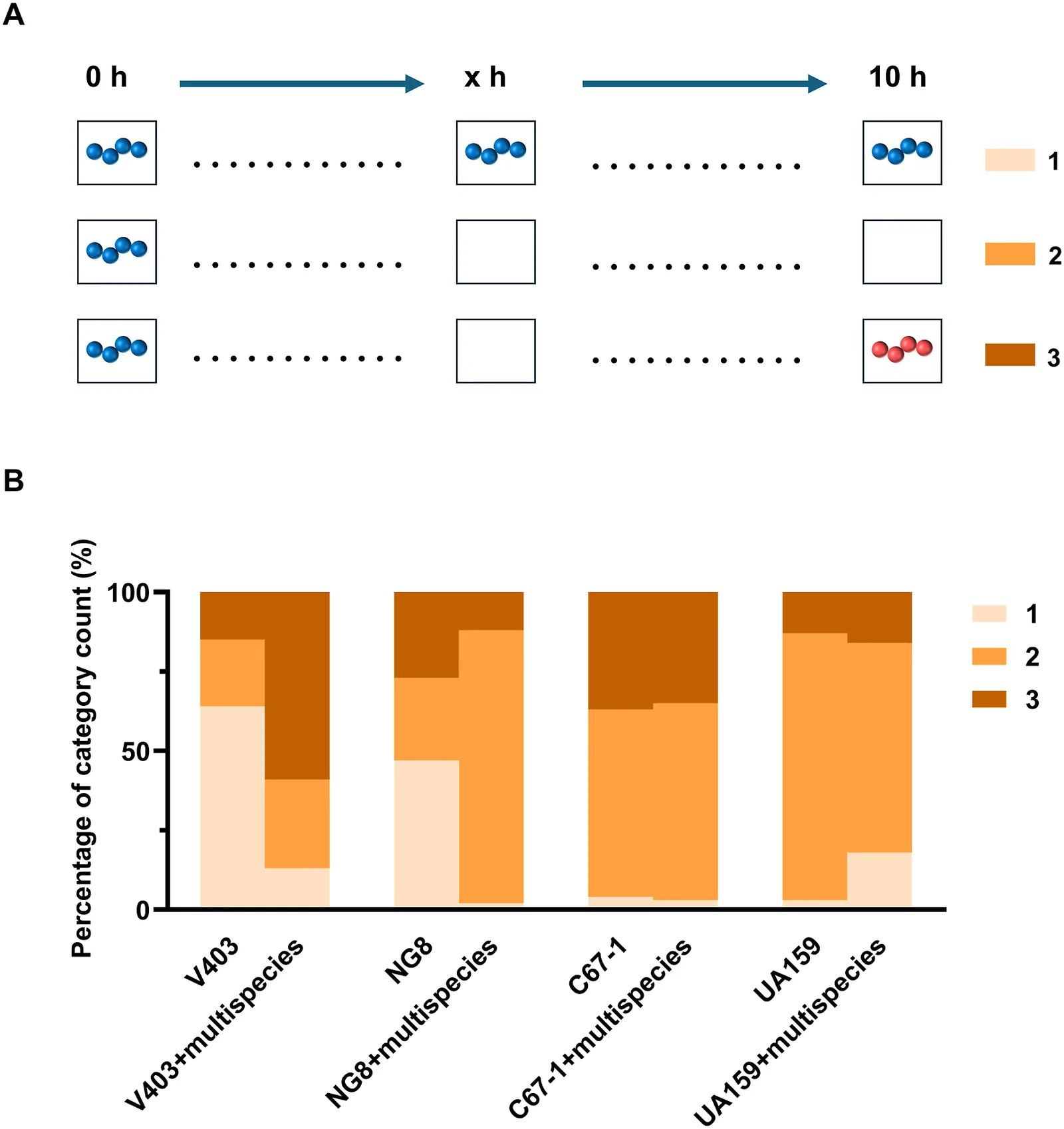
**Strain-specific biofilm displacement under crude salivary agglutinin (cSAG) coating and laminar flow.** Biofilm displacement of *S. mutans* strains with or without a multispecies culture was monitored over 10 h. (**A**) Three categories of cluster displacement over time were defined: (1) Cells or clusters remained stationary; (2) Cells or clusters detached and were removed by the flow; (3) Cells or clusters detached and were removed by the flow, but new cells or clusters later moved into the same or a new position and remained there. (**B)** Distribution of each displacement category for each strain under single-species and multispecies conditions.

Based on displacement patterns, 3 scenarios were defined (Fig. 5A): (1) Cells or clusters remained stationary; (2) Cells or clusters detached and were removed by the flow; (3) Cells or clusters detached and were removed by the flow, but new cells or clusters later moved into the same or a new position and remained there. The displacement patterns associated with biofilm development for each strain were then categorized according to these scenarios over the 10-h period, and the scenario distributions are summarized in Fig. 5B. The distribution patterns showed pronounced strain dependence and were substantially influenced by the presence of multispecies communities. For V403, more than 50% of clusters remained stationary in single-species culture, whereas the presence of multispecies community reduced this fraction, with a greater proportion of clusters being replaced by newly arriving clusters. NG8 behaved similarly to V403 under single-species conditions, although its cluster formation was less stable. In the presence of multispecies culture, NG8 clusters were removed more frequently. For C67-1, only a small fraction of clusters remained stationary over the 10-h period, regardless of whether multispecies were present. UA159 clusters or cells were predominantly removed under flow; however, the presence of multispecies increased the retention of clusters compared with single-species culture.

## 4 Discussion

In this study, the adherence and biofilm formation of four *S. mutans* strains were evaluated on cSAG-coated surfaces using the CBD and BioFlux™ systems under conditions that included salivary flow and the presence of a multispecies community, mimicking the oral cavity environment. Although initial adherence showed strain- and cSAG-dependent variation, these early attachment patterns alone were insufficient to explain the differences observed in subsequent biofilm formation. Instead, biofilm formation was shaped by the combined effects of strain-specific traits and environmental conditions, particularly the interaction between cSAG coating hydrodynamic forces and the presence of a microbial community. Laminar flow significantly enhanced the biofilm formation on cSAG-coated surfaces for all *S. mutans* strains, whereas the presence of a multispecies community altered biofilm formation in a manner dependent on hydrodynamic conditions, enhancing biofilm accumulation under static conditions but reducing it under flow. Together, these findings are consistent with previous reports that early attachment does not necessarily predict mature biofilm formation [5,6] and highlight the importance of considering dynamic environmental factors when investigating *S. mutans* biofilm formation.

Strain variability in biofilm formation has been widely reported for many bacterial species and is often attributed to differences in surface properties, adhesion mechanisms, and aggregation behavior [21,22]. In this study, strain-dependent differences in biofilm formation became apparent under flow conditions on cSAG-coated surfaces. V403 and NG8 showed increases of more than 200-fold under laminar flow compared to no-flow conditions, whereas UA159 showed an approximately 10-fold increase. These findings suggest that the combination of cSAG coating and hydrodynamic forces plays an important role in revealing strain-specific biofilm formation patterns.

Interestingly, the cSAG-mediated adhesion patterns reported in our previous study were not reproduced under the current experimental conditions. Whereas the strains were originally selected based on different adherence levels [12], three strains exhibited similar cSAG-mediated adhesion in the present study, while UA159 showed no detectable adhesion. The reason for this discrepancy remains unclear. Differences in the parotid saliva donors between the two studies may have contributed, as donor-dependent variation in cSAG properties, including SAG structural features (e.g. scavenger receptor cysteine-rich (SRCR) domain composition and glycosylation patterns), may influence adhesion. A previous study demonstrated that adherence to SAG containing 8 SRCR domains was approximately half of that observed for SAG containing 13 SRCR domains [23], suggesting that SAG structural variation can affect *S. mutans* adhesion. Further studies using cSAG prepared from multiple donors will be needed to determine how donor-dependent variation in cSAG properties influences strain-dependent adhesion patterns and whether it affects subsequent biofilm formation.

Although the role of SAG coating in mediating *S. mutans* adhesion is well established [24], its impact on subsequent biofilm formation remains unclear. Ahn et al. [25] reported that *S. mutans* biofilm formation was inhibited on saliva-coated surfaces and speculated that other salivary components may inhibit later stages of biofilm maturation, although SAG contributes to the initial stages of adherence and biofilm formation. In contrast, our results demonstrated that the crude SAG coating significantly enhanced biofilm formation, but only under laminar flow conditions. One possible explanation for this difference is that, under flow conditions, salivary components with potential biofilm-inhibitory effects may be removed from the surface, allowing the biofilm-promoting effect of cSAG to become more pronounced.

Beyond endpoint measurements to biofilm formation, such as surface coverage and cluster counts, the time-series analysis provided insight into the dynamics of strain-dependent biofilm development. The cluster formation (Fig. 4) and displacement dynamics (Fig. 5) revealed that strains with similar endpoint biomass could follow distinct developmental patterns. For example, although V403 and NG8 reached comparable biomass after 10 h, their cluster dynamics differed, with NG8 exhibiting substantially lower cluster stability under flow, whereas V403 formed stable clusters that persisted throughout biofilm development. This difference may contribute to the pronounced reduction in NG8 biofilm formation in the presence of a multi-species community. Consistent with this interpretation, the supplementary video shows that although NG8 formed substantial biomass through the rapid accumulation of numerous small clusters under single-species conditions, newly formed clusters were rapidly displaced under multi-species conditions, preventing the establishment of a stable biofilm. Previous studies have demonstrated that naturally occurring bacterial aggregates, rather than individual cell attachment, can serve as the primary units of biofilm initiation and strongly influence subsequent biofilm development [26,27]. Our findings extend this concept by showing that, beyond the initial formation of aggregates, their subsequent persistence under flow is strain dependent and may influence biofilm development within a multi-species community. Further investigation of the molecular mechanisms contributing to strain-dependent differences in cluster formation and retention may provide new insight into the factors governing biofilm persistence and removal under flow conditions.

Flow conditions are also known to influence biofilm formation through multiple mechanisms. Flow can promote biofilm growth by enhancing nutrient transport to the biofilm surface and facilitating diffusion within the matrix [28]. In addition, low flow rates can allow the local accumulation of quorum sensing (QS) molecules, which could further stimulate biofilm formation [29,30]. In our experiments, we applied a laminar flow of 0.15 dyn/cm^2^, which approximates the shear stress exerted by resting saliva, and observed enhanced biofilm formation for most strains compared with static conditions. Such slow flow has been shown to promote the accumulation of QS molecules, such as autoinducer-2 (AI-2), particularly in multispecies communities [31], thereby enhancing both biofilm biomass and aggregate size in multispecies biofilms, effects that are often absent in mono-species biofilms [32,33]. This mechanism may help explain the large aggregates observed for V403 when grown with a multispecies community. However, QS-mediated biofilm enhancement alone cannot account for the reduced V403 biofilm biomass under multispecies conditions. Our track-ID analysis (Fig. 5) indicated that the large aggregates were more readily removed by flow, providing a plausible explanation for the reduced biomass.

This study also examined adhesion and biofilm formation in two experimental models: the CBD and the BioFlux™ microfluidic system. The CBD provides a simple and high-throughput platform but is limited by its static nature and inability to capture biofilm architecture and flow-dependent features [34]. In contrast, the BioFlux™ provides controlled flow and enables real-time imaging of biofilm development under dynamic conditions, allowing better representation of the oral environment. However, its constrained throughput and reliance on costly disposable flow plates limit its broader application [35]. Under the conditions evaluated in this study, strain-dependent cSAG-mediated adhesion and no-flow biofilm formation showed similar patterns between the two systems, suggesting that differences observed under flow conditions may reflect responses to hydrodynamic conditions rather than solely model-specific effects.

A limitation of this study is that the saliva-derived multispecies biofilm could not be reliably visualized. We initially attempted to label the microbial community using fluorescent DNA-binding dyes, but staining interfered with real-time monitoring of *S. mutans* biofilm dynamics. Consequently, imaging was restricted to brightfield microscopy, in which the low intrinsic contrast of individual bacterial cells made them difficult to distinguish from the background. Hence, the present study focused primarily on the behavior of GFP-labeled *S. mutans*. A second limitation is the relatively short biofilm growth duration (10 h), constrained by the BioFlux™ system. Microfluidic flow chambers are generally optimized for early biofilm development, as prolonged growth can lead to extensive channel colonization and complicate maintenance of controlled flow and nutrient conditions. A further limitation is the use of cSAG preparation, which may contain a mixture of salivary components and therefore does not allow the observed effects to be attributed exclusively to SAG itself. Previous studies have demonstrated that cSAG preparations retain functional activity in supporting *S. mutans* adhesion [7], and our previous biochemical characterization demonstrated enrichment of SAG in this preparation [36]. However, contributions from other salivary components cannot be excluded. Therefore, the findings of this study should be interpreted as the response of *S. mutans* to a cSAG-coated surface rather than to a purified SAG coating. Further mechanistic studies would be valuable to define the specific contribution of SAG to these observations.

## 5 Conclusions

In conclusion, this study demonstrates that variation in initial cSAG-mediated adherence among *S. mutans* strains was not sufficient to predict subsequent biofilm formation. Instead, biofilm formation was shaped by multiple interacting factors, including strain-specific characteristics, environmental conditions such as laminar flow and sucrose availability, and the presence of a microbial community. Importantly, cSAG-coated surfaces not only promoted *S. mutans* attachment but also enhanced its biofilm formation under flow conditions. These findings underscore the importance of using experimental systems that incorporate physiologically relevant conditions to investigate biofilm dynamics and guide future strategies for biofilm control.

## CRediT authorship contribution statement

**Chen Sun:** Data curation, Formal analysis, Investigation, Software, Validation, Visualization, Writing – original draft. **Danuta Mazurel:** Data curation, Formal analysis, Investigation, Software, Validation. **Jingmei Yang:** Investigation, Methodology. **Antonius J.M. Ligtenberg:** Conceptualization, Resources, Writing – review & editing. **Dongmei Deng:** Conceptualization, Funding acquisition, Methodology, Project administration, Resources, Supervision, Writing – review & editing.

## Funding

C. Sun was supported by a scholarship from the 10.13039/501100004543China Scholarship Council. The BioFlux 1000Z microfluidic system was funded by 10.13039/501100013956NWO Earth and Life Sciences.

## Declaration of competing interests

The authors declare the following financial interests/personal relationships which may be considered as potential competing interests: Chen Sun reports financial support was provided by China Scholarship Council. The BioFlux 1000Z microfluidic system was funded by NWO Earth and Life Sciences If there are other authors, they declare that they have no known competing financial interests or personal relationships that could have appeared to influence the work reported in this paper.

## Data Availability

The original contributions presented in the study are included in the article/Supplementary Material. Further inquiries can be directed at the corresponding authors.
