# Supplementary material for: From adhesion to biofilm: strain-specific and environmental factors shaping Streptococcus mutans biofilms
Source: Biofilm. 2026 Aug 17;12:100391. doi: 10.1016/j.bioflm.2026.100391 (PMC13520613; doi:10.1016/j.bioflm.2026.100391)
Supplement: Supplementary file 1 [file mmc1.docx]

Supplementary Data

**Figure S1**. Biofilm formation of single *S. mutans* strains in 0.2% sucrose. Biofilms were grown under laminar flow without SAG coating and quantified at 10 h as percentage of surface coverage.

A

B

C

D

**Figure S2**. Representative images of *S. mutans* biofilm formation on SAG-coated surfaces under laminar flow.

Images were acquired using a 10x objective and a FITC filter (Filter set 44, Zeiss). Excitation was at 488 nm (bandwidth 30 nm) and emission at 520 nm (bandwidth 40 nm), with a 150 ms exposure time for all groups. Biofilms were imaged at 1, 3, 7, and 10 h, without (left panel) or with multispecies community (right panel). A. V403; B. NG8; C. C67-1; D. UA159. Scale bar: 50 µm.

**Supplementary video** Inhibition of multispecies community on *S. mutans* NG8 biofilm development under laminar flow.

Videos were recorded using a 10x Zeiss objective at 30 min intervals over 10 h. GFP-labelled NG8 was visualized using a FITC filter (Filter set 44, Zeiss). Multispecies communities were not stained and were observed under bright-field imaging A. Single *S. mutans* NG8; B. *S. mutans* NG8 in the presence of a multispecies community. Scale bar: 50 µm.
